# Best-subset instrumental variable selection method using mixed integer optimization with applications to health-related quality of life and education–wage analyses

**DOI:** 10.21203/rs.3.rs-5550004/v1

**Published:** 2024-12-04

**Authors:** Muhammad Qasim, Kristofer Månsson, Narayanaswamy Balakrishnan

**Affiliations:** 1 Department of Economics, Finance and Statistics, Jönköping International Business School, Jönköping University, Sweden; 2 Department of Mathematics and Statistics, McMaster University, Hamilton, Ontario, Canada

**Keywords:** Causal inference, Instrumental variables, Lasso, Best-subset selection, Mixed integer programming, Variable selection, Sparse regression, C13, C26, C36

## Abstract

The classical best-subset selection method has been demonstrated to be nondeterministic polynomial-time hard and thus presents computational challenges. This problem can now be solved via advanced mixed integer optimization (MIO) algorithms for linear regression. We extend this methodology to linear instrumental variable (IV) regression and propose the best-subset instrumental variable (BSIV) method incorporating the MIO procedure. Classical IV estimation methods assume that IVs must not directly impact the outcome variable and should remain uncorrelated with nonmeasured variables. However, in practice, IVs are likely to be invalid, and existing methods can lead to a large bias relative to standard errors in certain situations. The proposed BSIV estimator is robust in estimating causal effects in the presence of unknown IV validity. We demonstrate that the BSIV using MIO algorithms outperforms two-stage least squares, Lasso-type IVs, and two-sample analysis (median and mode estimators) through Monte Carlo simulations in terms of bias and relative efficiency. We analyze two datasets involving the health-related quality of life index and proximity and the education–wage relationship to demonstrate the utility of the proposed method.

## 1. Introduction

This work introduces the best-subset selection (BSS) method for selecting valid and invalid instrumental variables (IVs) for the linear causal model. A valid instrument is only correlated with the endogenous variable, whereas an invalid instrument also directly affects the outcome (dependent) variable, making it invalid. BSS is, by many researchers, considered the “holy grail” of variable selection in sparse regression since it looks for a global optimum (see Hastie et al. (2020)). The issue with the traditional BSS is that it is nondeterministic polynomial-time (NP)-hard (Natarajan, 1995) and is therefore computationally infeasible in high-dimensional settings. In a recent study, Bertsimas et al. (2016) used a mixed-integer optimization (MIO) technique to address the problem of the classical BSS method for linear regression. According to their findings, the best-subset problem can be solved efficiently to achieve certifiable global optimality via highly optimized MIO solvers such as Gurobi. In this paper, we extend for the first time the framework of Bertsimas et al. (2016) in the context of IVs. MIO for BSS uses the property that the classical best-subset problem is nonconvex. When a statistical method involves nonconvex optimization, it is possible to obtain a reformulation of the computational challenge so that it can be solved efficiently to achieve certifiable global optimality via highly optimized MIO solvers. Since the introduction of MIO for BSS by Bertsimas et al. (2016), it has been used in numerous studies; for example, see Sato et al. (2016), Mazumder & Radchenko (2017), Chen & Lee (2018), Takano & Miyashiro (2020), Hazimeh & Mazumder (2020), Saishu et al. (2021) and Wang et al. (2023). To our knowledge, no study has examined the use of MIO for solving the classical BSS problem in the linear IV regression model.

Glymour et al. (2012) reported that the validity of instruments is often violated in practice, resulting in invalid instruments and severely biased IV estimates. Research on identifying invalid instruments for high-dimensional data has been quite extensive in recent years. Kang et al. (2016) proposed a Lasso-type procedure for two-stage least squares (TSLS), which is computationally as fast as ordinary least squares (OLS). Windmeijer et al. (2019) introduced the adaptive Lasso estimator, which is consistent when fewer than 50% of the instruments are invalid. Guo et al. (2018) and Windmeijer et al. (2021) proposed confidence interval methods for estimating the causal effect in the case of invalid instruments. Lin et al. (2024) introduced robust IV estimation methods to address cases with many weak and invalid instruments. Moreover, median (Bowden et al., 2016) and mode (Hartwig et al., 2017) estimators have been used to perform accurate causal inference without selecting valid and invalid instruments, especially in the context of Mendelian randomization methods from the meta-analysis literature.

This study makes two main contributions. First, we generalize mixed integer linear programming to apply the BSS approach to estimate sparse linear IV models that contain unknown invalid IVs. In addition, we develop discrete first-order algorithms to find the optimal solution for the best-subset IV problem. Second, by using Monte Carlo simulation and real-life applications, we provide extensive empirical comparisons of the best-subset method with other widely used one-sample and two-sample summary methods under different conditions. The simulation results demonstrate that the novel best-subset IV estimator (BSIVE) with the MIO procedure performs very well relative to all other estimators in terms of bias and median squared error (MSE). We also use two real-life datasets to demonstrate the benefits of the proposed method. The first real-life analysis is based on Mendelian randomization, wherein we estimate the causal effect of depression and body mass index on health-related quality of life, using single-nucleotide polymorphisms (SNPs) as instruments for body mass index. The second application estimates the causal effect of education (years of schooling) on wages (log of earnings) by using college proximity and parents’ education as potential instruments. In addition, to simplify the implementation of the proposed method, we developed an R package called bsive(), which is also provided here.

The remainder of this paper is organized as follows. In Section 2, we present the causal model and its estimation methods with valid and invalid instruments. MIO implementation for the best-subset IV approach is introduced in Section 3. We introduce first-order methods in convex optimization to obtain near-optimal solutions for the best-subset IV problem in Section 4. The theoretical properties and performance of the proposed method are discussed in Section 5. We show in the Monte Carlo simulation study that the proposed BSIVE yields substantial improvements over existing estimators in terms of bias, MSE, and relative efficiency, as described in Section 6. The benefits of the BSIVE are also demonstrated through two real-life applications in Section 7. Finally, some concluding remarks are made in Section 8.

## 2. Causal model and estimation methods

Let $Y_{i}$ represent the observed outcome for the $i^{th}$ individual, where the exposure variables are denoted by $X_{i}$ and the vector of L potential instruments are represented by $Z_{i}$. Additionally, we denote the subset size as r, which ranges from 0 to min(n,L). Suppose that the variables $\left(Y_{i},X_{i}^{⊤},Z_{i}^{⊤}:i=1,\ldots ,n\right)$ are independently and identically distributed, and the model of interest is as follows:

(2.1) $$Y_{i}=X_{i}^{⊤}\beta^{0}+Z_{i}^{⊤}\delta^{0}+\epsilon_{i}\;X_{i}=\psi^{0^{⊤}}Z_{i}+\mu_{i},$$

where $Y_{i}$ is a scalar, $X_{i}$ is an m×1 vector of endogenous (exposure) variables, $E\left[\epsilon_{i}|Z_{i}\right]=0,\beta^{0}\in R^{m},\delta^{0}\in R^{L},\psi^{0}={\left[E\left(Z_{i}Z_{i}^{⊤}\right)\right]}^{-1}E\left[Z_{i}X_{i}^{⊤}\right]$ are the true parameters and $E\left[\mu_{i}|Z_{i}\right]=0$. The parameter of interest is the causal parameter vector, $\beta^{0}$. The values of $\delta^{0}\;$ indicate valid and invalid instruments. The definition of valid and invalid instruments states that the instruments (j=1,…,L) are valid when $\delta_{j}^{0}=0$ and that the instruments (j=1,…,L) are invalid when $\delta_{j}^{0}\neq 0$. The definition of a valid instrument corresponds to the formal definition in Holland (1988) and Kang et al. (2016). For any full-rank matrix $Z\in R^{\left(n\times L\right)},M_{Z}=I-P_{Z}$ is the residual-forming matrix, where $P_{Z}=Z{\left(Z^{⊤}Z\right)}^{-1}Z^{⊤}$ is the projection matrix onto the column space of Z and where I is an identity matrix of dimensions n×n. The $l_{p}$-norm is denoted by $\|\cdot {\|}_{p}$, so the $l_{0}$-norm corresponds to $\|\cdot {\|}_{0}$, which shows the number of nonzero components of a vector; the $l_{\infty }$-norm is denoted by $\|\cdot {\|}_{\infty }$, which indicates the maximum element of a vector. It is desirable to assume that the parameter vector δ is known to be r-sparse.

### 2.1 Oracle two-stage least squares

The oracle model, represented in matrix form, is defined as $Y=X\beta^{0}+Z_{in}\delta_{in}^{0}+\epsilon$, where $Z_{in}$ is the $\left(n\times L_{in}\right)$ set of invalid instruments and where $\delta_{in}^{0}$ represents the coefficient vector of the invalid instruments. The first-stage regression is then given as $X=Z\psi^{0}+\mu =Z_{va}\psi_{va}^{0}+Z_{in}\psi_{in}^{0}+\mu$, where $Z_{va}$ is an $\left(n\times L_{va}\right)$ matrix of valid IVs. Furthermore, we have the following assumptions:

1. $\left(Y_{i},X_{i}^{⊤},Z_{i}^{⊤}:i=1,\ldots ,n\right)$ are independently and identically distributed;
2. $E[Y{]}^{4}<\infty ,E\|X{\|}^{4}<\infty$, and $E\|Z{\|}^{4}<\infty$;
3. $E\left(\epsilon_{i}^{2}|Z_{i}\right)=\sigma_{\epsilon }^{2}$ and $\Sigma =\left[\begin{array}{cc} \sigma_{\epsilon }^{2} & \sigma_{\epsilon \mu }\\ \sigma_{\mu \epsilon } & \sigma_{\mu }^{2} \end{array}\right]$ is finite, and
4. $\text{rank}\left[\psi_{va}^{0}\right]=m$ and $\Sigma_{\mu \mu }$ is positive definite.

Assumption 1 is a basic assumption stating that the observations are i.i.d. Assumption 2 states that the dependent variable, exposure, and instruments have finite fourth moments. In assumption 3, we first make a conditional homoscedasticity assumption on the error given the instruments, and we next assume that the elements of Σ are finite (Guo et al., 2018). Assumption 4 identifies the coefficients of the endogenous variables, and the full rank condition on $\Sigma_{\mu \mu }$ is equivalent to supposing that X and Z have no common elements. The oracle TSLS (OTSLS) method is a method in which the matrix $Z_{in}$ is known and is given by

$${\overset{ˆ}{\theta }}_{\text{OTSLS}}=\left[\begin{array}{c} \overset{ˆ}{\beta }\\ {\overset{ˆ}{\delta }}_{in} \end{array}\right]={\left({𝒲}^{⊤}P_{Z}𝒲\right)}^{-1}{𝒲}^{⊤}P_{Z}Y,$$

where $𝒲=\left[\begin{array}{ll} X & Z_{in} \end{array}\right]$. Under assumptions 1–4, when n→∞, the asymptotic distribution of OTSLS is given by

$$n^{1/2}\left({\hat{\theta }}_{\text{OTSLS}}-\theta \right)\underset{\to }{d}𝒩\left(0,V_{\theta }\right),$$

where $V_{\theta }=\sigma_{\epsilon }^{2}{\left(Q_{XZ}Q_{ZZ}^{-1}Q_{ZX}\right)}^{-1},Q_{XZ}=E\left[X_{i.}Z_{i.}^{⊤}\right],Q_{ZZ}=E\left[Z_{i}Z_{i}^{⊤}\right],Q_{ZX}=E\left[Z_{i.}X_{i.}^{⊤}\right]$, and $\sigma_{\epsilon }^{2}$ is the variance of the error term ϵ.

### 2.2 Lasso-type IV approach

In observational studies, IVs can directly affect the outcome variable. Conventional estimation methods may require comprehensive knowledge about the validity of all instruments; a valid instrument should neither directly impact the outcome nor be correlated with the unmeasured confounding variables. However, in certain circumstances, this comprehensive knowledge may be lacking. To address this issue, Kang et al. (2016) introduced a Lasso-type IV estimator (LIVE) by using convex optimization methods (S. S. Chen et al., 2001; Tibshirani, 1996) to select and identify the set of invalid instruments. Furthermore, Windmeijer et al. (2019) and Lin et al. (2024) focused their research on identifying unknown invalid IVs via a modified version of the Lasso procedure. The LIVE is based on the $l_{1}$ norm and selects the IV that is directly associated with the outcome variable. Kang et al. (2016) modified the Lasso procedure of Tibshirani (1996) for the linear IV model with one endogenous variable. The LIVE can identify valid instruments and estimate the causal effect under the weak condition that the proportion of invalid instruments is strictly less than 50% of the total instruments even without prior knowledge of the instrument’s validity. By convex duality, the LIVE is given in penalized form as

(2.2) $${\hat{\beta }}^{\left(\lambda \right)},{\hat{\delta }}^{\left(\lambda \right)}\in \text{arg}\;\underset{\beta ,\delta }{\text{min}}\frac{1}{2}{\left\|{\mathbb{P}}_{Z}\left(Y-X\beta -Z\delta \right)\right\|}_{2}^{2}+\lambda \|\delta {\|}_{1},$$

where λ≥0 is the tuning parameter estimated through cross-validation by minimizing the estimated equation ${\left\|P_{Z}(Y-X\beta -Z\delta )\right\|}_{2}$. A fast two-step algorithm is suggested to estimate the solution to problem (2.2), which is as computationally fast as ordinary least squares. The optimization problem in (2.2) is different from those in the work of Belloni et al. (2012) and Spindler (2016) in terms of the penalty for the parameters in the model. Problem (2.2) penalizes only δ, whereas other problems penalize the causal effect of the endogenous variables on the output variable by assuming that either all IVs are valid or we know exactly which subset of them are valid. Kang et al. (2016) noted that the best-subset problems in linear IV models are computationally infeasible and NP-hard. To address this issue, we propose a novel BSIVE method using advanced MIO in the following section.

## 3. MIO implementation for the best-subset IV approach

In this section, we first extend the classical BSS procedure to linear IV models. Next, we provide a brief overview of the mixed integer linear programming technique. We then introduce the MIO implementation for the best-subset IV problem.

### 3.1 Best-subset selection

The literature on estimating a classical sparse linear model with good explanatory power has naturally increased in the last few decades. The IV method is a traditional technique for estimating causal effects. Here, we extend the classical best-subset approach in the IV framework. However, the validity of the instruments is suspect in many situations, even in high-dimensional settings. For example, consider the case of Mendelian randomization^1^, where a genetic marker, which is an SNP, is used as an instrument. SNPs (one or more of the genes may have multiple targets) might have a direct effect on the outcomes or be associated with unmeasured confounders. In this situation, it is necessary to select the instruments that have a direct effect on the outcome variable. We aim to find the best r-feature fit to the outcome variable. The best-subset problem for r, which is stated as the following optimization problem, is fundamental to this statistical task:

(3.1) $$\underset{\beta ,\delta }{\text{min}}\frac{1}{2}{\left\|{\mathbb{P}}_{Z}\left(Y-X\beta -Z\delta \right)\right\|}_{2}^{2}\;\text{subject}\;\text{to}\;\|\delta {\|}_{0}\leq r,$$

where $\|\delta {\|}_{0}=\sum_{j=1}^{L}1\left(\delta_{j}\neq 0\right)$ with indicator function 1(⋅) and subset size r⊆{1,…,L}.

We present a two-step procedure for solving the best-subset problem in (3.1). Let $\overset{ˆ}{X}=P_{Z}X,P_{\overset{ˆ}{X}}=P_{Z}X{\left(X^{⊤}P_{Z}X\right)}^{-1}X^{⊤}P_{Z},M_{\overset{ˆ}{X}}=I-P_{\overset{ˆ}{X}}$, and $P_{\overset{ˆ}{X}}P_{Z}=P_{\overset{ˆ}{X}}$. Then, optimization problem (3.1) can be written as

$$\underset{\beta ,\delta }{\text{min}}\frac{1}{2}{\left\|{\mathbb{P}}_{\hat{X}}\left(Y-Z\delta \right)-\hat{X}\beta \right\|}_{2}^{2}+\frac{1}{2}{\left\|M_{\hat{X}}{\mathbb{P}}_{Z}\left(Y-Z\delta \right)\right\|}_{2}^{2}\;\text{subject}\;\text{to}\;\|\delta {\|}_{0}\leq r.$$

For any given $\delta \in R^{L}$, the expression $\frac{1}{2}{\left\|P_{\overset{ˆ}{X}}(Y-Z\delta )-\overset{ˆ}{X}\beta \right\|}_{2}^{2}$ becomes zero because $P_{\overset{ˆ}{X}}(Y-Z\delta )$ lies in the range of $\overset{ˆ}{X}$, and hence, we define the best-subset regression problem equivalently as

(3.2) $$\hat{\delta }\in \text{arg}\;\underset{\delta }{\text{min}\;}f\left(\delta \right)\;\text{subject}\;\text{to}\;\|\delta {\|}_{0}\leq r,$$

where $f(\delta )=\frac{1}{2}\|\dot{Y}-\dot{Z}\delta {\|}_{2}^{2},\dot{Y}=M_{\overset{ˆ}{X}}P_{Z}Y$ and $\dot{Z}=M_{\overset{ˆ}{X}}Z$. Then, we estimate $\overset{ˆ}{\beta }$ via $\overset{ˆ}{\delta }$ from (3.2) as

(3.3) $$\hat{\beta }={\left({\hat{X}}^{⊤}\hat{X}\right)}^{-1}{\hat{X}}^{⊤}\left(Y-Z\hat{\delta }\right).$$

The cardinality constraints mean that optimization problem (3.1) is NP-hard. Owing to the computational NP-hardness of $\|\delta {\|}_{0}$ in the linear model, Tibshirani (1996) proposed a surrogate penalty function that was later extended by Kang et al. (2016) and Windmeijer et al. (2019) in the linear IV model. For many years, a fundamental belief in statistics has been that the best-subset selection problem is typically limited to a maximum number of regressors, such as 30. For example, a well-known statistical package in R, called leaps(), does not handle problems with more than 30 regressors. Bertsimas et al. (2016) showed that this problem can now be solved via advanced MIO for classical linear regression. Therefore, we use the modern MIO technique to find a provably optimal solution for the BSS optimization problem in the linear IV model.

### 3.2 Overview of MIO

We start by giving a general brief overview of MIO, a mixed-integer quadratic programming problem that is also an optimization problem, as follows:

$$\text{min}\;\alpha^{⊤}H\alpha +c^{⊤}\alpha$$

s.t.α∈𝒫,

$$\alpha_{i}\in \left\{0,1\right\},\;\forall i\in 𝒥,$$

$$\alpha_{j}\geq 0,\;\forall j\in 𝒥,$$

where the quadratic coefficient matrix is $H\in R^{q\times q},\;c\in R^{q},\;𝒫$ is a polyhedron with $𝒫=\{\alpha :A\alpha \leq b\},A\in R^{r\times q}$, and $b\in R^{r}$, and we optimize $\alpha \in R^{q}$ containing both discrete and continuous variables with 𝒥⊆{1,…,q}. We recommend Bertsimas & Weismantel (2005) and Bertsimas et al. (2016) for background details on MIO.

### 3.3 Instrumental variable regression via MIO

We now show that the BSS approach in (3.2) can be modeled as an MIO. Let $w={\left(w_{1},\ldots ,w_{L}\right)}^{⊤}$ be the binary variable and big-ℳ>0 be an arbitrary number such that $ℳ\geq \|\overset{ˆ}{\delta }{\|}_{\infty }$, where $\overset{ˆ}{\delta }$ is a minimizer of ${\text{min}}_{\left(\delta ,w\right)}\frac{1}{2}\|Y-Z\delta {\|}_{2}^{2}$ subject to $-ℳw_{i}\leq \delta_{i}\leq ℳw_{i}$ and i=1,…,L. Note that the parameter ℳ is not subject to statistical tuning, as we have the flexibility to select any sufficiently large value. When $w_{k}=1$, the only possibility is to make $-ℳ\leq \delta_{i}\leq ℳ$, and if $w_{i}=0$, then $\delta_{i}=0$. Therefore, $\sum_{i=1}^{L}w_{i}$ is an indicator of the number of nonzero elements in δ. More formally, the best-subset instrumental variables (BSIV) problem in (3.2) can be formulated as a mixed integer quadratic optimization (MIQO) problem as follows:

(3.4) $$\underset{\delta ,w}{\text{min}}\frac{1}{2}\delta^{⊤}\left(Z^{⊤}M_{\hat{X}}Z\right)\delta -\langle \delta^{⊤}M_{\hat{X}}{\mathbb{P}}_{Z}Y,\delta \rangle +\frac{1}{2}Y^{⊤}{\mathbb{P}}_{Z}M_{\hat{X}}{\mathbb{P}}_{Z}Y$$

(3.5) $$\text{subject}\;\text{to}\;w_{i}=0\Rightarrow \delta_{i}=0,\;\forall i\in \left[L\right],$$

(3.6) $$w_{i}\in \left\{0,1\right\},\;\forall i\in \left[L\right]$$

(3.7) $$\underset{i\in \left[L\right]}{\sum }w_{i}\leq r,$$

(3.8) $$-{ℳ}_{i}\leq \delta_{i}\leq {ℳ}_{i},\;\forall i\in \left[L\right],$$

(3.9) $${ℳ}_{1i}\geq \|\delta {\|}_{1},$$

where the logical expression in (3.5) can be modeled by a special ordered set type-1 (SOS1) constraint with the method of Beale & Tomlin (1970) and can be written as $\left\{1-w_{i},\delta_{i}\right\}=\text{SOSI}$ ∀i∈[L]. The objective function in (3.4) requires specification of the desired sparsity, denoted by r, on the right-hand side of (3.7). The constraints (3.6) and (3.8) ensure the values of $w_{i}=1$ if $\delta_{i}\neq 0$ and $w_{i}=0$ if $\delta_{i}=0.\;{ℳ}_{i}$ and ${ℳ}_{1i}\in [0,\infty ]$ yield upper bounds on |δ| and $\|\overset{ˆ}{\delta }{\|}_{1}$, respectively. Any sufficiently large value of ℳ can result in a solution for optimization problem (3.2); thus, the performance of MIO depends on these bounds, as discussed in Section 3.4. The bounds can be calculated via various methods on the basis of the data. However, while the bounds on δ may not be needed, if these constraints are provided, this will increase the robustness of the MIO formulation; see, e.g., Hazimeh & Mazumder (2020).

Remark 1: The MIQO formulations in Eqs. (3.4)–(3.9) have a quadratic form in the IVs, which is more useful in the setting n>L, and we expand these formulations for the high-dimensional setting L≪n. Now, problem (3.4) can be modified as follows:

$$\underset{\delta ,w,\xi }{\text{min}}\frac{1}{2}\xi^{⊤}\xi -\langle Z^{⊤}M_{\hat{X}}{\mathbb{P}}_{Z}Y,\delta \rangle +\frac{1}{2}Y^{⊤}{\mathbb{P}}_{Z}M_{\hat{X}}{\mathbb{P}}_{Z}Y$$

$$\text{subject}\;\text{to}\;\;\xi =ZM_{\hat{X}}\delta ,$$

$$\underset{i\in \left[L\right]}{\sum }w_{i}\leq r,$$

$$-w_{i}{ℳ}_{i}^{\delta }\leq \delta_{i}\leq w_{i}{ℳ}_{i}^{\delta },\;{ℳ}_{1i}^{\delta }\geq \|\delta {\|}_{1},$$

$$-w_{i}{ℳ}_{i}^{\xi }\leq \xi_{i}\leq w_{i}{ℳ}_{i}^{\xi },\;{ℳ}_{1i}^{\xi }\geq \|\xi {\|}_{1},$$

$$w={\left\{0,1\right\}}^{L},\;\delta \in {\mathbb{R}}^{L},\;\xi \in {\mathbb{R}}^{n}.$$

### 3.4 Parameter specifications for δ

The restriction on $\delta_{i}$ is given by ${ℳ}_{i}\in [L]$ in (3.8). We can calculate the bounds on $\delta_{i}$, which are based simply on the data-driven method of performing convex quadratic optimization. Considering the setting n>L, which is very common in Mendelian randomization analysis, economics, and some other fields, we obtain the upper bounds (UBs) and lower bounds (LBs) on ${\overset{ˆ}{\delta }}_{i}$ by solving the following problems:

(3.10) $$h_{i}^{-}=\underset{\delta }{\text{min}}\;\delta_{i}\;\text{s}.\text{t}\;\frac{1}{2}{\left\|M_{\hat{X}}\left({\mathbb{P}}_{Z}Y-Z\delta \right)\right\|}_{2}^{2}\leq \text{UB},\;h_{i}^{+}=\underset{\delta }{\text{max}\;}\delta_{i}\;\text{s.t}\;\frac{1}{2}{\left\|M_{\hat{X}}\left({\mathbb{P}}_{Z}Y-Z\delta \right)\right\|}_{2}^{2}\leq \text{UB,}$$

where $h_{i}^{-}$ and $h_{i}^{+}$ are the UB and LB of $\delta_{i}\forall i\in [L]$, respectively, and the right-hand side of (3.10) is a UB of minimization problem (3.2). We consider $h_{i}^{-}$, which is a minimization problem; using the framework of quadratic optimization (Bertsimas et al., 2016; Boyd & Vandenberghe, 2004), it can be deduced that there exists an η such that

(3.11) $$f^{\prime }\left(\frac{1}{2}{\left\|M_{\hat{X}}\left({\mathbb{P}}_{Z}Y-Z\delta \right)\right\|}_{2}^{2}+\eta \delta_{i}\right)=0,$$

where we assume without loss of generality that the parameter δ satisfies the condition that the gradient (3.11) remains feasible for (3.10) and that $f^{\prime }(\cdot )$ is the derivative with respect to δ. We can express (3.11) in a simplified form as

$$\delta ={\left(Z^{T}M_{\overset{ˆ}{X}}Z\right)}^{-1}\left(Z^{⊤}M_{\overset{ˆ}{X}}P_{Z}Y+\eta \tau_{i}\right),$$

where the ith coordinate of $\tau_{i}\in R^{L}$ is equal to one, while the rest are zero. To find the optimal value that solves (3.10), the above results in (3.11) are used to obtain

$${\left\|M_{\hat{X}}\left({\mathbb{P}}_{Z}Y-Z\delta \right)\right\|}_{2}^{2}={\left\|M_{\hat{X}}{\mathbb{P}}_{Z}Y-M_{\hat{X}}Z{\left(Z^{T}M_{\hat{X}}Z\right)}^{-1}M_{\hat{X}}{\mathbb{P}}_{Z}Y-M_{\hat{X}}Z{\left(Z^{T}M_{\hat{X}}Z\right)}^{-1}\eta \tau_{i}\right\|}_{2}^{2}.$$

The expression ${\left\|M_{\overset{ˆ}{X}}\left(P_{Z}Y-Z\delta \right)\right\|}_{2}^{2}=\text{UB}$ clearly shows that the parameter η satisfies the quadratic results mentioned above. Therefore, this optimal value solves the problem in (3.10). Similarly, $h_{i}^{+}={\text{min}}_{\delta }\delta_{i}$ can be solved by changing it from a maximization problem to a minimization problem, such as $-h_{i}^{+}={\text{min}}_{\delta }-\delta_{i}$ such that $\frac{1}{2}{\left\|M_{\overset{ˆ}{X}}\left(P_{Z}Y-Z\delta \right)\right\|}_{2}^{2}\leq \text{UB}$. Now, consider the UB of $\left|\delta_{i}\right|$ to be $-{ℳ}_{i}\leq \delta_{i}\leq {ℳ}_{i},\forall i\in [L]$, where ${ℳ}_{i}$ can be used as $\text{max}\left\{\left|h_{i}^{-}\right|,\left|h_{i}^{+}\right|\right\}$, as given in (3.10).

## 4. Optimal solutions for BSIV problems

In this section, we extend the discrete versions of first-order techniques in convex optimization to obtain near-optimal solutions for the BSIV problem. We establish its convergence analysis by building upon the framework introduced by Nesterov (2013, 2018), Bertsimas et al. (2016) and Bottou et al. (2018). This framework implements the idea of projected gradient descent methods in first-order convex optimization problems and simplifies them to solve the following optimization problem:

(4.1) $$\underset{\delta }{\text{min}}\;f\left(\delta \right)\;\text{s}.\text{t}\;\|\delta {\|}_{0}\leq r,$$

where the objective function f(⋅) is called Lipschitz continuous, with the Lipschitz constant $\left(L_{c}\right)$, for all $\{\delta ,\bar{\delta }\}\in R^{L}$,

$$\|f(\delta )-f(\bar{\delta }){\|}_{2}\leq L_{c}\|\delta -\bar{\delta }{\|}_{2}.$$

The function f(⋅) has a derivative (gradient) $f^{\prime }(\cdot )$, which is Lipschitz continuous with $L_{c}>0$. Then, f(δ) itself is called an $L_{c}$-smooth convex function and is expressed as

(4.2) $${\left\|f^{\prime }\left(\delta \right)-f^{\prime }\left(\bar{\delta }\right)\right\|}_{2}\leq L_{c}^{0}\|\delta -\bar{\delta }{\|}_{2}$$

where $f^{\prime }(\delta )=-Z^{⊤}M_{\overset{ˆ}{X}}\left(P_{Z}Y-Z\delta \right)$ and $L_{c}^{0}=\lambda_{\text{max}}\left(Z^{⊤}M_{\overset{ˆ}{X}}Z\right)$, with $\lambda_{\text{max}}(\cdot )$ being the largest eigenvalue of the given data matrix. The constraint in (4.2) is fundamental for the convergence analyses of the majority of gradient-based methods; without this constraint, the gradient cannot reliably reveal the direction or extent of movement required to decrease the objective function. By using the results of (4.2), we obtain the important inequality in Proposition 4.1 (Bottou et al., 2018; Nesterov, 2018).

**Proposition 4.1.** If f(δ) *is continuously differentiable and the gradient function is Lipschitz continuous with* $L_{c}\geq L_{c}^{0}$, *then we have the following inequality*:

(4.3) $$f\left(\delta \right)-f\left(\bar{\delta }\right)\leq f^{\prime }{\left(\bar{\delta }\right)}^{⊤}\left(\delta -\bar{\delta }\right)+\frac{1}{2}L_{c}\|\delta -\bar{\delta }{\|}_{2}^{2},\;\forall \delta ,\bar{\delta }\in {\mathbb{R}}^{L}.$$

*Proof of Proposition 4.1:* The objective function f(δ) is Lipschitz continuous with $L_{c}>0$. Therefore, we can obtain the following results via constraint (4.2) with $f(\tau )=f(\bar{\delta }+\tau (\delta -\bar{\delta })):$

$$f\left(\delta \right)-f\left(\bar{\delta }\right)=\int_{0}^{1}\frac{\partial f\left(\bar{\delta }+\tau \left(\delta -\bar{\delta }\right)\right)}{\partial \tau }d\tau \;=f^{\prime }{\left(\bar{\delta }\right)}^{⊤}\left(\delta -\bar{\delta }\right)+\int_{0}^{1}{\left[f^{\prime }\left(\bar{\delta }+\tau \left(\delta -\bar{\delta }\right)\right)-f^{\prime }\left(\bar{\delta }\right)\right]}^{⊤}\left(\delta -\bar{\delta }\right)d\tau \;\leq f^{\prime }{\left(\bar{\delta }\right)}^{⊤}\left(\delta -\bar{\delta }\right)+\int_{0}^{1}L_{c}\|\tau \left(\delta -\bar{\delta }\right){\|}_{2}\|\delta -\bar{\delta }{\|}_{2}d\tau .$$

We can also write the bound in (4.3) as a function of ${𝒬}_{r}(\delta )$ and then solve this bound subject to $\|\bar{\delta }{\|}_{2}\leq r$ as

$$f(\bar{\delta })\leq {𝒬}_{r}(\delta )≔\frac{1}{2}L_{c}\|\bar{\delta }-\delta {\|}_{2}^{2}+f^{\prime }(\delta {)}^{⊤}(\bar{\delta }-\delta )+f(\delta ).$$

With a current solution δ, the proposed method minimizes the UB to $f(\bar{\delta })$ around f(δ). To show this, we draw upon the notions from projected gradient descent methods in first-order convex optimization problems and apply Proposition 4.1:

(4.4) $$\underset{\|\bar{\delta }{\|}_{0}\leq r}{\text{min}}{𝒬}_{r}\left(\delta \right)\;=\underset{\|\bar{\delta }{\|}_{0}\leq r}{\text{min}}\left(\frac{1}{2}L_{c}{\left\|\bar{\delta }-\left(\delta -\frac{1}{L_{c}}f^{\prime }\left(\delta \right)\right)\right\|}_{2}^{2}\right)\;={𝒮}_{r}\left(\delta -\frac{1}{L_{c}}f^{\prime }\left(\delta \right)\right).$$

One fundamental element in solving the above problem is implementing (Donoho & Johnstone, 1994) the hard-thresholding operator, ${𝒮}_{r}(\cdot )$. The hard-threshold rule is similar to the rules used in model selection for selecting variables. Bertsimas et al. (2016) reported that $\overset{ˆ}{\delta }$ retains the r greatest elements of any $\theta \in R^{L}$ and sets the remaining elements to zero if $\overset{ˆ}{\delta }$ is an optimal solution to the following optimization problem:

(4.5) $$\overset{ˆ}{\delta }\in \text{arg}\underset{\|\delta {\|}_{0}\leq r}{\text{min}}\|\delta -\theta {\|}_{2}^{2}.$$

The entries in θ are ordered as follows: $\left|\theta_{\left(1\right)}\right|\geq \left|\theta_{\left(2\right)}\right|\geq \cdots \geq \left|\theta_{\left(L\right)}\right|$. The hard-thresholding operator, ${𝒮}_{r}(\theta )$, of problem (4.4) has the following form:

(4.6) $${\overset{ˆ}{\delta }}_{i}=\left\{\begin{array}{ll} \theta_{i}, & \;\text{if}\;i\in \{(1),(2),\ldots ,(r)\}\\ 0, & \;\text{otherwise}. \end{array}\right.$$

With the results of (4.4), the discrete first-order optimization method performs the following updates until a convergence criterion is met:

(4.7) $$\text{Iteration:}\;\delta^{\left(t+1\right)}\in {𝒮}_{r}\left(\delta^{\left(t\right)}-\frac{1}{L_{c}}f^{\prime }\left(\delta^{\left(t\right)}\right)\right).$$

The algorithm is formally outlined below for calculating the estimate $\overset{ˆ}{\delta }$:

**Table:** 

**Algorithm 1** : Discrete first order
**Require:** $\dot{Y}$: response vector; $\dot{Z}$: model matrix; $L_{c}$*; tol:* convergence tolerance
1: Start with $\delta^{\left(1\right)}$ that is r-sparse;
2: Obtain $\delta^{\left(t+1\right)}$, for iteration t≥1, as $$\;\delta^{\left(t+1\right)}\in {𝒮}_{r}\left(\delta^{\left(t\right)}-\frac{1}{L_{c}}f^{\prime }\left(\delta^{\left(t\right)}\right)\right);$$
3: Repeat update Step 2 until $\;{\left\|\delta^{\left(t+1\right)}-\;\delta^{\left(t\right)}\right\|}_{2}^{2}\leq \text{tol}$;
4: **Output**: Return the regression coefficients.

Our method for conducting the convergence analysis for a first-order stationary solution, denoted by ${\overset{ˆ}{\delta }}^{f}$, from the output of algorithm 1, is built upon the smoothness result of the objective function.

**Theorem 4.1.** *Under constraints* (4.2) *and* (4.3), *the iterates of the first-order method* (Algorithm 1) *satisfy the following results for* $L_{c}\geq L_{c}^{0}$ *after* T *iterations*:

$$\underset{1\leq t\leq T}{\text{min}}{\left\|\delta^{\left(t+1\right)}-\delta^{\left(t\right)}\right\|}_{2}^{2}\leq \frac{2}{T\left(L_{c}-L_{c}^{0}\right)}\left(f\left(\delta^{\left(1\right)}\right)-f\left({\overset{ˆ}{\delta }}^{f}\right)\right),$$

*where* $f\left(\delta^{\left(1\right)}\right)$ *is decreasing and converges to some* $f\left({\overset{ˆ}{\delta }}^{f}\right)$ *as* t→∞.

*Proof of Theorem 4.1*: The convergence rate of Algorithm 1, which is based on the modified outcome variable and design matrix for the linear IV model, follows from the results in Theorem 3.1 of Bertsimas et al. (2016).□

Theorem 4.1 shows that using the discrete first-order method to solve the optimization problem in (4.1) results in a decreasing sequence of objective values, which eventually converges. In $T=O\left(\frac{1}{\text{tol}}\right)$ iterations, where tol>0, it follows that ${\left\|\delta^{\left(t^{f}+1\right)}-\delta^{\left(t^{f}\right)}\right\|}_{2}^{2}\leq \text{tol}$.

## 5. Properties of the BSIV estimator

We study the statistical properties of the proposed estimator and its performance in estimating the causal effect. In addition, a theoretical comparison of the problem in (3.2) with the Lasso procedure is conducted. Consider the model (2.1) with one endogenous variable and $\overset{ˆ}{\delta }$ to be a solution to the problem in (3.2) via the discrete first-order algorithm. Given the $l_{0}$-based best-subset estimator, $\overset{ˆ}{\delta }$, we estimate^2^ the causal effect as

(5.1) $$\overset{ˆ}{\beta }=\text{arg}\;\underset{\beta }{\text{min}}{\left\|P_{\overset{ˆ}{X}}\left(Y-Z\overset{ˆ}{\delta }\right)-\overset{ˆ}{X}\beta \right\|}_{2}^{2}.$$

**Lemma 5.1.** *Suppose that we have the model* $Y=Z\delta^{0}+\epsilon$, *where* $\delta^{0}$ *is* r-sparse,r∈[L], *and we let* $\overset{ˆ}{\delta }$ *be a solution to the best-subset problem. Define* $\Delta (r)=\left\{\zeta \in R^{L}:\|\zeta {\|}_{0}\leq r\right\}$, *and determine the error bound*, ${𝒜}_{r}=\left\{\epsilon^{T}Z\zeta \leq \sigma {\left(5r\;\text{log}(eL/r)+\text{log}(r/L{)}^{-r}\right)}^{1/2}\right\}$. *Then, we have the following bounds with a probability of at least* $1-(r/L{)}^{r}$:

- *The prediction error is given by*
$$\|Z\overset{ˆ}{\delta }-Z\delta {\|}_{2}^{2}≲\sigma^{2}r\;\text{log}(eL/r),$$
- *and the estimation error is given by*
$$\|\overset{ˆ}{\delta }-\delta {\|}_{2}^{2}≲\frac{\sigma^{2}r\;\text{log}(eL/r)}{(\eta (r){)}^{2}},$$
  *where* $\eta \left(r\right)=\text{min}\underset{\zeta \in \Delta \left(r\right)}{\zeta \neq 0}\frac{\left\|Z\zeta \right\|}{\left\|\zeta \right\|}$ .

*Proof of Lemma 5.1*: *We define the error bounds*

$${𝒜}_{r}=\left\{\epsilon^{⊤}Z\zeta \leq \sigma {\left(5r\;\text{log}(eL/r)+\text{log}(r/L{)}^{-r}\right)}^{1/2}\right\}$$

by using deterministic reasoning on appropriately chosen random events by following the results of Raskutti et al. (2011) and Mazumder et al. (2023). By the assumed observed model, $Y=f_{z}+\epsilon$, where $f_{z}=Z\delta^{0}$ denotes the unknown deterministic mean and δ∈Δ(r), we have the following results:

$${\left\|f_{z}-Z\overset{ˆ}{\delta }\right\|}_{2}^{2}\leq {\left\|f_{z}-Z\delta \right\|}_{2}^{2},$$

which implies that

(5.2) $$\|Y-Z\overset{ˆ}{\delta }{\|}_{2}^{2}\leq \|Y-Z\delta {\|}_{2}^{2}+2\epsilon^{⊤}Z\left(\overset{ˆ}{\delta }-\delta \right).$$

Now, by controlling the expression $\epsilon^{⊤}Z(\overset{ˆ}{\delta }-\delta )$, we establish the fast prediction error rate for the estimator $\overset{ˆ}{\delta }$. We have the following result for the error terms using event ${𝒜}_{r}$:

$$\epsilon^{⊤}Z\left(\overset{ˆ}{\delta }-\delta \right)\leq \sigma {\left(5r\;\text{log}(eL/r)+\text{log}(r/L{)}^{-r}\right)}^{\frac{1}{2}}\left\|Z\left(\delta -\overset{ˆ}{\delta }\right)\right\|,$$

which suggests that

(5.3) $${\left\|f_{z}-Z\hat{\delta }\right\|}_{2}^{2}\leq {\left\|f_{z}-Z\delta \right\|}_{2}^{2}+2\sigma {\left(5r\;\text{log}\left(eL/r\right)+\text{log}{\left(r/L\right)}^{-r}\right)}^{1/2}\left(\left\|f_{z}-Z\hat{\delta }\right\|+\left\|f_{z}-Z\delta \right\|\right).$$

The second expression on the right-hand side of the above inequality is bounded by applying the following inequity. Let a,b∈R and the parameter θ>0. Then, we have

$$2ab\leq \theta a^{2}+\frac{b^{2}}{\theta }.$$

Let θ=4; we find the desired inequalities via the above results:

$$2\sigma {\left(5r\;\text{log}\left(eL/r\right)+\text{log}{\left(r/L\right)}^{-r}\right)}^{1/2}\left(\left\|f_{z}-Z\hat{\delta }\right\|+\left\|f_{z}-Z\delta \right\|\right)\;\leq 2\sigma^{2}\left(5r\;\text{log}\left(eL/r\right)+\text{log}{\left(r/L\right)}^{-r}\right)+{\left(\left\|f_{z}-Z\hat{\delta }\right\|+\left\|f_{z}-Z\delta \right\|\right)}^{2}$$

and

$$\sigma {\left(r\;\text{log}\left(eL/r\right)+\text{log}{\left(r/L\right)}^{-r}\right)}^{1/2}\left(\left\|f_{z}-Z\hat{\delta }\right\|+\left\|f_{z}-Z\delta \right\|\right)\;≲\sigma^{2}r\;\text{log}\left(eL/r\right)+\sigma^{2}\;\text{log}{\left(r/L\right)}^{-r}+{\left(\left\|f_{z}-Z\hat{\delta }\right\|+\left\|f_{z}-Z\delta \right\|\right)}^{2}$$

By considering inequality (5.3), we determine the fast-rate prediction error bound as

(5.4) $${\left\|f_{z}-Z\overset{ˆ}{\delta }\right\|}_{2}^{2}≲\underset{\delta \in \Delta (r)}{\text{inf}}{\left\|f_{z}-Z\delta \right\|}_{2}^{2}+\sigma^{2}r\;\text{log}(eL/r)+\sigma^{2}\;\text{log}(r/L{)}^{-r}.$$

We have a result focused on $\overset{ˆ}{\delta }$, as well as the above fast prediction error bounds on the random constraint ${𝒜}_{r}$. Hence, the prediction error (a) and the estimation error bound (b) are derived by setting $f_{z}=Z\delta^{0}$ for some $\delta^{0}\in \Delta (r)$ in the above results with a probability of at least $1-(r/L{)}^{r}$:

$${\left\|Z\overset{ˆ}{\delta }-Z\delta^{0}\right\|}_{2}^{2}≲\sigma^{2}r\;\text{log}(eL/r).$$

Then, we have the results for the estimation error bounds, which are obtained from the inequality $(\eta (r){)}^{2}{\left\|\overset{ˆ}{\delta }-\delta^{0}\right\|}_{2}^{2}\leq {\left\|Z\overset{ˆ}{\delta }-Z\delta^{0}\right\|}_{2}^{2}$.□

The prediction error and estimation error bounds are shown in Lemma 5.1. The right-hand sides of the above inequalities depend on the noise variance of the model and the r-sparse model. The unknown parameter, $\sigma^{2}$, is estimated by fitting the regression model, and the parameter r is tuned via cross-validation. The results of Lemma 5.1 are useful for determining the theoretical performance of the causal effect estimator based on Eq. (5.1). The estimated difference between $\overset{ˆ}{\beta }$ and $\beta^{0}$, i.e., $\left|\overset{ˆ}{\beta }-\beta^{0}\right|$, is analyzed to examine the theoretical performance of the BSIV estimator. Theorem 5.1 shows the performance of the proposed estimator.

**Theorem 5.1.** *Let the observed data model be* $Y_{i}=X_{i}\beta^{0}+Z_{i}^{⊤}\delta^{0}+\epsilon_{i}$ *under assumptions 1–4, and let* $\overset{ˆ}{\beta }$ *be the causal estimator of* β. *Then, the estimated causal effect*, $\overset{ˆ}{\beta }$, *under the BSIV with the MIO framework has the following performance guarantee*:

$$\left|\overset{ˆ}{\beta }-\beta^{0}\right|\leq \frac{1}{\|\overset{ˆ}{X}{\|}_{2}}\left(\frac{\sigma (r\;\text{log}(eL/r){)}^{\frac{1}{2}}}{{\left(\eta^{*}\left(r\right)\right)}^{2}}\right)+\frac{\left|{\overset{ˆ}{X}}^{⊤}\epsilon \right|}{\|\overset{ˆ}{X}{\|}_{2}^{2}},$$

*where* $\eta^{*}(r)=\text{min}\underset{\zeta \in \Delta (r)}{\zeta \neq 0}\frac{\left\|P_{\overset{ˆ}{X}}Z\zeta \right\|}{\left\|\zeta \right\|}$.

*Proof of Theorem 5.1:* The optimization problem in (3.1) can be estimated via two-step numerical algorithms. The first step is to obtain the best subset with MIO, and the second step is to conduct the simple least-squares estimation in (5.1). We modify the model $Y_{i}=X_{i}\beta^{0}+Z_{i}^{⊤}\delta^{0}+\epsilon_{i}$, in lieu of Eq. (3.2), by applying Lemma 5.1 as follows:

$$\dot{Y}=\dot{Z}\delta^{0}+\dot{\epsilon }$$

where $\dot{Y}=M_{\overset{ˆ}{X}}P_{Z}Y,\;\dot{Z}=M_{\overset{ˆ}{X}}Z$ and $\;\dot{\epsilon }=M_{\overset{ˆ}{X}}P_{Z}\epsilon$. On the basis of the modified model, we have the following result:

$${\dot{\epsilon }}^{⊤}\dot{Z}\left(\overset{ˆ}{\delta }-\delta \right)\leq \sigma {\left(5r\;\text{log}(eL/r)+\text{log}(r/L{)}^{-r}\right)}^{\frac{1}{2}}\left\|M_{\overset{ˆ}{X}}Z\left(\delta -\overset{ˆ}{\delta }\right)\right\|,$$

which implies, by Lemma 5.1(a), that

(5.5) $${\left\|M_{\hat{X}}Z\left(\hat{\delta }-\delta^{0}\right)\right\|}_{2}≲\sigma^{2}r\;\text{log}\left(eL/r\right).$$

The estimator $\overset{ˆ}{\beta }$ in Eq. (3.1) using $\overset{ˆ}{\delta }$ with the MIO framework has the following form:

$$\overset{ˆ}{\beta }=\beta^{0}-\frac{1}{\|\overset{ˆ}{X}{\|}_{2}^{2}}\left(\left({\overset{ˆ}{X}}^{⊤}P_{\overset{ˆ}{X}}\overset{ˆ}{Z}\left(\overset{ˆ}{\delta }-\delta^{0}\right)\right)+{\overset{ˆ}{X}}^{T}P_{\overset{ˆ}{x}}\epsilon \right)$$

Applying norms to both sides of the equation and simplifying the terms, we obtain

(5.6) $$\left|\overset{ˆ}{\beta }-\beta^{0}\right|\leq \frac{{\left\|P_{\overset{ˆ}{X}}Z\left(\overset{ˆ}{\delta }-\delta^{0}\right)\right\|}_{2}}{\|\overset{ˆ}{X}{\|}_{2}}+\frac{\left|{\overset{ˆ}{X}}^{⊤}\epsilon \right|}{\|\overset{ˆ}{X}{\|}_{2}^{2}}.$$

The bound on the estimation error $\overset{ˆ}{\delta }-\delta^{0}$ means that the numerator of the first term on the right-hand side of the inequality in (5.6) is necessary to bound $\left|\overset{ˆ}{\beta }-\beta^{0}\right|$. From the results of Lemma 5.1, we establish the results for the estimation error in (5.5), which allows us to convert the bound for $|P_{\overset{ˆ}{X}}Z\left(\overset{ˆ}{\delta }-\delta^{0}\right){\|}_{2}$ to establish the following result:

(5.7) $${\left\|\overset{ˆ}{\delta }-\delta^{0}\right\|}_{2}≲\frac{\sigma }{\eta^{*}(r)}(r\text{log}(eL/r){)}^{2},$$

where $\eta^{\text{*}}\left(r\right)=\text{min}\underset{\zeta \in \Delta \left(r\right)}{\zeta \neq 0}\frac{\left\|{\mathbb{P}}_{\hat{X}}Z\zeta \right\|}{\left\|\zeta \right\|}$ is the minimal r-sparse eigenvalue of $P_{\overset{ˆ}{X}}Z$. Finally, using the results of (5.7) in (5.6), we obtain the desired bound. □

### 5.1 Risk bounds for the BSIVE and LIVE

In the framework of IV regression, the LIVE aims to estimate β and δ with sparsity in δ. In the context of sparsity in δ, the estimation error bounds for the LIVE can be defined by following Bühlmann & Van De Geer (2011), such as ${\left\|{\overset{ˆ}{\delta }}_{\text{L}}-\delta^{0}\right\|}_{2}^{2}=o\left(\frac{r\;\text{log}(L)}{n}\right)$, where r represents the number of nonzero elements in the sparsity of δ and the parameter vector ${\overset{ˆ}{\delta }}_{\text{L}}$ gives the Lasso-based solutions. We now compare the asymptotic risk of BSIVE with that of LIVE. Zhang et al. (2014) and Bertsimas et al. (2016) defined the predictive risk of BSS in the context of linear regression, and we extend this definition to the linear IV regression framework. Accordingly, BSIVE satisfies the following upper bounds.

(5.8) $$\underset{\beta ;\delta^{0}:{\left\|\delta^{0}\right\|}_{0}\leq r}{\text{max}}\frac{1}{n}E\left(\|P_{Z}X\left(\overset{ˆ}{\beta }-\beta^{0}{\|}_{2}^{2}\right)≲\frac{\sigma^{2}(r\;\text{log}(L)+n\cdot \text{rank}(Z))}{n}.\right.$$

Now, we focus on the LIVE penalty, which shows that δ is sparse. Therefore, the $\ell_{1}$-norm bound on δ allows us to control ${\left\|Z\left(\delta -\delta^{0}\right)\right\|}_{2}^{2}$. The matrix Z satisfies the restricted eigenvalue (RE) condition w.r.t. S⊆{1,…,L} if $\frac{1}{n}{\left\|Z\left(\delta -\delta^{0}\right)\right\|}_{2}^{2}\geq \eta \left(Z\right){\left\|\delta -\delta^{0}\right\|}_{2}^{2}\;\;\forall \delta \in C(S)$, where $C(S)=\left\{\delta :\sum_{j\notin S}\left|\delta_{j}\right|\leq 3\sum_{j\in S}\left|\delta_{j}\right|\right\}$. Since the parameter of interest is β, we state that the matrix $\overset{ˆ}{X}=P_{Z}X$ satisfies the RE condition if

$$\frac{1}{n}{\left\|P_{Z}X\left(\beta -\beta^{0}\right)\right\|}_{2}^{2}\geq \eta \left(P_{Z}X\right){\left\|\beta -\beta^{0}\right\|}_{2}^{2},$$

where $\eta (\overset{ˆ}{X})$ is the RE constant. Suppose that ${\overset{ˆ}{\beta }}^{\lambda }$ and ${\overset{ˆ}{\delta }}^{\lambda }$ solve problem (2.2) and that ${\overset{ˆ}{\beta }}^{r(\lambda )}$ and ${\overset{ˆ}{\delta }}^{r(\lambda )}$ are their threshold versions. ${\overset{ˆ}{\delta }}^{r(\lambda )}$ keeps the top r elements (invalid instruments) of ${\overset{ˆ}{\delta }}^{\lambda }$ and sets the others to zero, which yields the number of valid instruments. By using RE conditions, we obtain the following results:

(5.9) $$\frac{\sigma^{2}\left(r^{1-t}\;\text{log}\left(L\right)+n\cdot \text{rank}\left(Z\right)\right)}{\eta {\left({\hat{X}}_{\text{bad}}\right)}^{2}n}≲\underset{\beta ;\delta^{0}:{\left\|\delta^{0}\right\|}_{0}\leq r}{\text{max}}\frac{1}{n}E(\|{\mathbb{P}}_{Z}X\left({\hat{\beta }}^{r\left(\lambda \right)}-\beta^{0}{\|}_{2}^{2}\right)\;≲\frac{\sigma^{2}\left(r\;\text{log}\left(L\right)+n\cdot \text{rank}\left(Z\right)\right)}{\eta {\left(\hat{X}\right)}^{2}n},$$

where the lower bounds hold for poorly constructed design matrices ${\overset{ˆ}{X}}_{\text{bad}}={\left(P_{Z}X\right)}_{\text{bad}}$ for any arbitrarily small scalar t>0 Bertsimas et al. (2016). Eq. (5.8) and Eq. (5.9) show that a significant gap exists in the predictive performance between the BSIVE- and LIVE-based r sparse solutions. This difference can be especially large when $\eta (\overset{ˆ}{X})$ is small. The LIVE introduces bias in the regression coefficient estimates due to the $\ell_{1}$-norm penalty, and it does not consistently select the invalid instrument (Windmeijer et al., 2019). On the other hand, when the BSIVE identifies valid and invalid instruments, it does so without applying any shrinkage to the coefficients, thereby reducing the influence of the relative strength of the instruments.

## 6. Simulation study

We examine the finite-sample behavior of the proposed estimators through Monte Carlo simulations. We first obtain a model by following the data-generating process:

(6.1) $$Y=X\beta^{0}+Z\delta^{0}+\epsilon \;X=Z\psi^{0}+\mu ,$$

where the instrumental variables Z are drawn from the multivariate normal distribution, i.e., $Z\overset{\text{i.i.d}}{\sim }𝒩\left(0,\Sigma_{z}\right)$, where $\Sigma_{z}$ has entries (j,k) equal to $\rho^{\left|j-k\right|},\;\rho$ is a correlation between instruments, $\sigma_{\mu }^{2}=1$, and $\sigma_{\mu \epsilon }=0.25$. By following Kang et al. (2016), we consider four different values ρ=0, 0.25, 0.50 and 0.75, representing varying degrees of correlation between the instruments (i.e., from no correlation to strong correlation). We set the parameter value $\beta^{0}=0$, the value of $\psi^{0}$ to 0.20, and $\delta^{0}={\left(0.20_{0.3L},0_{0.7L}\right)}^{⊤}$, with 30% of the instruments being invalid (Windmeijer et al., 2019); the total number of instruments corresponds to L=10 and 40 in $\left\|\delta^{0}\right\|$. We choose the case of L=40 to show that BSIVE can be used with any set of IVs and is no longer an NP-hard problem, unlike BSS. The causal parameter $\beta^{0}$ is the main quantity of interest. Windmeijer et al. (2019) derived the signal-to-noise ratio (SNR) for a specific model (5.8), which is given by

$$\text{SNR}=\frac{\delta^{0^{⊤}}{𝒬}_{11}\delta^{0}}{\sigma_{\epsilon }^{2}}$$

where ${𝒬}_{11}$ is an r×r matrix from $𝒬=\text{plim}\left(n^{-1}{\dot{Z}}^{⊤}\dot{Z}\right)$ and $\dot{Z}=M_{\overset{ˆ}{X}}Z$. On the basis of Hastie et al. (2020), we consider five values, SNR = 0.10, 0.25, 1, 2 and 6. This range covers values from low to high SNRs, and the proportions of variance explained are given as 0.09, 0.20, 0.50, 0.67 and 0.85, respectively. The tuning parameter λ for LIVE is selected through 10 -fold cross-validation (Kang et al., 2016). Selecting λ through cross-validation is a very common data-driven approach in the literature that aims for optimal prediction accuracy. Takano & Miyashiro (2020) established the MIO approach for selecting the best subset via the cross-validation criterion and used 10-fold cross-validation in the simulation setting. The choice of the subset size through cross-validation and its computational cost are also discussed in Hastie et al. (2020). In all the cases, tuning is performed by minimizing the prediction error when using 10-fold cross-validation. The parameter of interest is $\beta^{0}$, which sets the moment condition $E\left(Z^{⊤}\left(Y-X\beta^{0}-Z\delta^{0}\right)\right)=0$. We use the R package sisVIVE(), which is available on CRAN, to estimate the LIVE results. For convenience, we created an R package called bsive() for implementing the proposed method via the Gurobi MIO solver.

The Monte Carlo simulation results are presented in terms of bias, MSE, and relative efficiency (RE) over 1000 replications under one– and two–sample analyses. These one– and two–sample analyses were discussed in Burgess et al. (2023): one–sample methods can be used when IVs, endogenous variables, and outcomes are measured on the same individuals, whereas two–sample methods can be used when IVs and endogenous associations are measured in one dataset and IVs and outcome associations are estimated in a second dataset. The performance of the proposed method, BSIVE, is compared with that of several other methods: one-sample information methods, including OLS, oracle–TSLS (OTSLS), naive-TSLS (NTSLS), and LIVE, and two–sample methods, namely, median (Bowden et al., 2016) and mode (Hartwig et al., 2017) estimators^3^. OTSLS is naturally preferred because we assume precise knowledge of valid and invalid instruments. However, in real-life scenarios, identifying exactly which instruments are valid and invalid is challenging. The NTSLS estimator assumes that all instruments are valid and is therefore a naive benchmark. In this study, we introduce the BSIVE estimator to address situations where knowledge of the validity of instruments is lacking. We aim for our method’s performance to approximate that of the optimal OTSLS. Minelli et al. (2021) examined the performance of both two-sample (median and mode) and one-sample methods for Mendelian randomization analyses and reported that the assumption that the samples are homogenous may be violated in practice. One-sample Mendelian randomization can be used when the two-sample method is not feasible (Denault et al., 2022). Therefore, we include median and mode estimators to assess robustness.

### 6.1 Simulation results

Tables 1–5 present the Monte Carlo simulation results in terms of the bias and MSE of the methods for all values of the SNR, sample size (n) and correlation level between instruments (ρ). We calculate the RE as a performance measure to measure the improvement of the BSIVE over the existing one. The RE, expressed as a percentage, is defined as the ratio of the MSE of any existing estimator to that of the proposed estimator. Four different levels of ρ are given in Tables 1–4 for cases in which the number of instruments (L) is ten with three invalid instruments (r: nonzero entries). The simulation results indicate that the BSIVE with the MIO procedure performs very well across all the cases. In almost all the scenarios evaluated in terms of MSE, the BSIVE performs the best. It is only when ρ=0,n=200 and the SNR=0.1 in Table 1 that the Mode estimator outperforms BSIVE in terms of MSE. Hence, when ρ≥0.25 in Tables 2–4, the BSIVE is superior to all estimators except the optimal OTSLS in terms of MSE. Regarding bias, we can see in Table 1 that the Mode estimator outperforms the BSIVE when n=200 for any value of SNR and for n=500 when SNR=0.1. It also outperforms the BSIVE in terms of bias in Table 2 when n=200 and the SNR equals 0.1 or 0.25 and for n=500 when the SNR equals 0.1. When the correlation corresponds to ρ=0.5 and ρ=0.75, the BSIVE is superior to all the estimators except OTSLS both in terms of bias and MSE. However, the MSE of the BSIVE is close to that of OTSLS. It also converges toward it as the sample size and SNR increase. This is a common finding in the literature (Kang et al., 2016; Lin et al., 2024), but the bias and MSE of the BSIVE are much closer to those of OTSLS than the LIVE estimator proposed by Kang et al. (2016). On the other hand, the NTSLS estimator, which assumes that all the instruments are valid, performs poorly, as it fails to address the bias introduced by invalid instruments. Overall, the simulation results indicate that the novel best-subset IV method with the MIO procedure outperforms other estimators.

The conventional optimization problem in (3.1) is NP-hard. For example, if we estimate the first-stage regression through a well-known method, leaps-and-bounds, it is only useful for L<30 (Moka et al., 2024). However, the BSIVE with the MIO algorithm can be used when L is much larger than 30. Table 5 summarizes the results for L=40 with r=12 and an SNR equal to one. The BSIVE performs best and has superior performance compared with all other estimators in terms of bias and MSE (Table 5). The NTSLS has the worst performance in all the cases, and OTSLS has the best performance. The BSIVE is usually very close to OTSLS, especially in terms of MSE. The conclusion of this simulation study is, therefore, that the BSIVE with the MIO procedure is an alternative robust choice for real-life scenarios where knowledge about the validity of instruments is lacking. The BSIVE often performs similarly to the OTSLS estimator; therefore, it should be considered when selecting valid and invalid instruments.

## 7. Real-life data analyses

In this section, we analyze two datasets to illustrate the utility of the proposed method^4^.

### 7.1 Dataset 1: Mendelian randomization analysis

One challenge in Mendelian randomization analysis related to the exclusion assumption is that some available instruments can be invalid, as they may directly affect the outcome of interest. This problem can be treated as a model selection problem if no prior knowledge exists regarding the validity of the instruments. We consider Mendelian randomization applications, in which we estimate the causal effect of depression and body mass index (BMI) on the health-related quality of life index (HRQLI) using SNPs as instruments. Gaynes et al. (2002) analyzed the impact of depression on health-related quality of life in a non-Mendelian randomization study. The study revealed a negative impact of depression on HRQLI. Sach et al. (2007) showed that BMI is negatively associated with HRQLI. However, a key challenge in these studies is that HRQLI includes various individual factors, which makes it difficult to account for all potential confounders affecting depression, BMI and HRQLI. This problem could be addressed through Mendelian randomization, which allows the control of unmeasured variables (Kang et al., 2016).

We use 5 SNPs^5^ (rs1800497, rs2242592, rs1421085, rs6314, and rs6265) as instruments for the exposure (depression) variable; the first four SNPs have been identified in genome-wide association studies (GWASs) (Hwang et al., 2006; Roetker et al., 2012). In the second case, we analyze the causal effect of BMI on HRQLI using 47 SNPs as instruments, which can be linked to the outcome variable to create a challenging scenario for the proposed methods to assess their robustness. HRQLI is estimated via the health utility index (HUI) developed by Horsman et al. (2003), which is a summary measure of several health attributes, such as vision, hearing, and cognitive skills. An HUI value of 1 indicates “perfect health”, and a value of 0 indicates a “dead” state. The HUI can be negative, which represents a state “worse than death” (Furlong et al., 2001; Molina et al., 2023). We use data from a Wisconsin longitudinal study, which include data of American high school graduates from Wisconsin who have been tracked since 1957. We study graduates who were genotyped in the survey data. We consider 95 SNPs as instruments for BMI in the raw dataset. After excluding missing observations, our analysis included 1816 observations and 47 SNPs. IVs (SNPs) may be invalid for various reasons, such as linkage disequilibrium, population stratification, and horizontal pleiotropy (Von Hinke et al., 2016; Windmeijer et al., 2019).

First, we test the validity of the instruments (Kang et al., 2021), and the J test rejects the null hypothesis that all the instruments are valid (p-value<0.001). We also verify that there is no significant correlation between IVs, which is a common situation in Mendelian randomization studies. The results of the causal effects of depression and BMI on HRQLI are presented in Table 6. Table 6 shows that the effects of depression and BMI on HRQLI are negative for all the estimators. The magnitudes of the estimates are low, and increasing depression and BMI by one unit can lead from a healthy state to a negative state (indicating that the person is alive but in a health state worse than death). When we analyze the impact of depression on HRQLI, the LIVE [−0.0062: 95% CI (−0.0071, −0.0051)] selects SNPs rs1800497, rs1421085 and rs6265 as invalid, whereas the BSIVE [−0.0046: 95% CI (−0.0064, −0.0027)] selects SNPs rs1800497, rs1421085, rs6314 and rs6265 as invalid. On the other hand, when we examine the effect of BMI on HRQLI, the BSIVE identifies 43 instruments as valid and 4 instruments as invalid, whereas the LIVE results indicate that 9 instruments are invalid. We also check the robustness of the instruments by using the F test. This shows that the TSLS might have a problem with weak instruments. It would be of interest to develop Lasso and BSS procedures with a robust estimator, for example, by using the (modified) limited information maximum likelihood optimization problem instead of TSLS; we plan to consider this in our future work. However, selecting valid and invalid instruments via the limited information maximum likelihood method is beyond the scope of this paper.

### 7.2 Dataset 2: Proximity and education–wage analysis

In this application, we use the dataset of Card (1993) to determine the causal effect of education on log earnings via four IVs. The dependent variable is the log hourly wage, and the endogenous variable, education, represents the highest level of schooling completed. Card (1993) considered binary IVs to determine whether the individual grew up in a place with a 2-year college and 4-year college nearby. In a recent study, Hou et al. (2020) also used either the mothers’ or fathers’ education as an IV. They observed that both parental education and individual schooling are positively correlated with wages, with one’s own education playing a more significant role in determining its effect than parental education does. In addition to college proximity, we use mothers’ and fathers’ education levels as IVs. The mothers’ and fathers’ education levels are significantly correlated, with ρ=0.63. The other IVs have correlation coefficient values between 0.08 and 0.15. The test of overidentification yields a value of 33.38, which indicates that the instruments used in the linear IV model are not valid (p-value<0.001). The F test statistic for the first-stage regression is equal to 193.11, which indicates that the model does not have a weak instrument problem. This shows that TSLS optimization is reasonable. The causal effects [$\overset{ˆ}{\beta }$: 95% CI] of education on the hourly wage for the OLS [0.0470: 95% CI (0.0402, 0.0538)], NTSLS [0.0747: 95% CI (0.0611, 0.0882)], Median estimator [0.0660: 95% CI (0.0405, 0.0915)] and Mode estimator [0.0620: 95% CI (0.0444, 0.0796)] are positive, indicating that, all else being equal, an increase in education by 1 year is associated with a corresponding increase in log earnings, on average. Both variable selection methods identify and select invalid instruments. The LIVE [0.0718: 95%CI(0.0685, 0.0752)] selects the two instruments of college proximity (nearc2: grew up near 2-yr college) and (nearc4: grew up near 4-yr college) as invalid, whereas the BSIVE [0.0553: 95%CI(0.0350, 0.0756)] identifies one instrument (mother’s education) as valid and college proximity and father’s education as being associated directly with wage and therefore invalid. Thus, the causal effect of education on wages when parental schooling is used as an IV is robust with the BSIVE. Therefore, using an optimal variable selection method can have an impact on the estimated causal effect.

## 8. Concluding Remarks

This work demonstrates that it is possible to determine and evaluate causal effects without a known set of valid instruments by using the best-subset problem with the modern MIO procedure. In addition, it is shown that the BSIVE method can solve the best-subset problem of choosing r out of L instruments in linear IV regression given n observations. Additionally, the novel BSIVE method outperforms other methods, including the LIVE, median, and mode estimators, in linear IV sparse models with possible invalid instruments. Therefore, the BSIVE is a robust choice for estimating causal effects. The usefulness of the proposed method is also demonstrated by using two real-life applications. One potential direction for future work is to expand the BSIVE framework to situations where the outcome variable is binary or the data are skewed. In such cases, a generalized linear model could be used. Additionally, if a large portion of the observations are zero, zero-inflated models may be more appropriate. We are currently working on these issues and hope to report the findings in a future paper.

## Figures and Tables

**Table 1:** Monte Carlo simulation results for estimation performance with ρ=0,L=10 and r=3.

Method	SNR = 0.10			SNR = 0.25			SNR = 1			SNR = 2			SNR = 6		
	Bias	MSE	RE (%)	Bias	MSE	RE (%)	Bias	MSE	RE (%)	Bias	MSE	RE (%)	Bias	MSE	RE (%)
*n* = 200															
OLS	0.2493	0.0621	38.8	0.2492	0.0621	17.7	0.2498	0.0624	8.2	0.2494	0.0622	5.6	0.2502	0.0626	4.2
NTSLS	0.2940	0.0864	27.9	0.2982	0.0890	12.4	0.2947	0.0868	5.9	0.2958	0.0875	4.0	0.2993	0.0896	2.9
Median	0.1897	0.0370	65.3	0.1644	0.0272	40.4	0.1261	0.0159	32.0	0.1107	0.0123	28.4	0.0936	0.0088	30.1
Mode	0.0745	0.0166	144.9	0.0538	0.0100	109.8	0.0519	0.0052	98.3	0.0462	0.0037	93.3	0.0508	0.0032	81.9
LIVE	0.2750	0.0756	31.9	0.2475	0.0613	17.9	0.1856	0.0345	14.8	0.1608	0.0259	13.5	0.1351	0.0183	14.4
BSIVE	0.1440	0.0241	100	0.0940	0.0110	100	0.0610	0.0051	100	0.0502	0.0035	100	0.0456	0.0026	100
OTSLS	0.0267	0.0069		0.0252	0.0044		0.0258	0.0024		0.0223	0.0017		0.0272	0.0014	
*n* = 500															
OLS	0.2504	0.0627	6.8	0.2515	0.0632	4.3	0.2510	0.0630	2.3	0.2497	0.0623	1.9	0.2505	0.0627	1.1
NTSLS	0.2944	0.0867	4.9	0.2964	0.0879	3.1	0.2996	0.0898	1.6	0.2980	0.0888	1.4	0.2951	0.0871	0.8
Median	0.1276	0.0164	25.9	0.1047	0.0111	24.4	0.0782	0.0063	23.3	0.0690	0.0048	25.2	0.0546	0.0030	23.8
Mode	0.0317	0.0066	64.6	0.0329	0.0045	60.5	0.0281	0.0023	64.1	0.0271	0.0018	67.7	0.0258	0.0011	66.0
LIVE	0.2051	0.0421	10.1	0.1612	0.0260	10.4	0.1176	0.0138	10.6	0.1040	0.0108	11.2	0.0836	0.0070	10.2
BSIVE	0.0345	0.0042	100	0.0267	0.0027	100	0.0234	0.0015	100	0.0213	0.0012	100	0.0208	0.0007	100
OTSLS	0.0164	0.0031		0.0094	0.0017		0.0088	0.0009		0.0122	0.0008		0.0111	0.0004	
*n* = 1000															
OLS	0.2491	0.0620	2.9	0.2509	0.0629	2.0	0.2499	0.0625	1.0	0.2496	0.0623	0.7	0.2501	0.0625	0.5
NTSLS	0.2985	0.0891	2.0	0.2984	0.0891	1.4	0.3002	0.0901	0.7	0.2975	0.0885	0.5	0.2987	0.0892	0.4
Median	0.0907	0.0084	21.2	0.0733	0.0055	23.3	0.0539	0.0029	20.8	0.0456	0.0021	22.4	0.0377	0.0014	23.9
Mode	0.0140	0.0037	48.3	0.0184	0.0024	53.2	0.0156	0.0011	52.8	0.0157	0.0008	61.8	0.0166	0.0006	53.9
LIVE	0.1392	0.0194	9.2	0.1150	0.0132	9.7	0.0840	0.0071	8.6	0.0703	0.0049	9.4	0.0571	0.0033	10.4
BSIVE	0.0129	0.0018	100	0.0165	0.0013	100	0.0129	0.0006	100	0.0108	0.0005	100	0.0121	0.0003	100
OTSLS	0.0048	0.0015		0.0064	0.0010		0.0063	0.0005		0.0042	0.0003		0.0063	0.0002	

Note: RE (%) represents the relative efficiency and is calculated by dividing the MSE of any estimator by that of the BSIVE. Oracle-TSLS (OTSLS) is the estimator used when we precisely know which instruments are valid and which ones are not. The performance of BSIVE approaches that of OTSLS.

**Table 2:** Monte Carlo simulation results for estimation performance with ρ=0.25,L=10 and r=3.

Method	SNR = 0.10			SNR = 0.25			SNR = 1			SNR = 2			SNR = 6		
	Bias	MSE	RE (%)	Bias	MSE	RE (%)	Bias	MSE	RE (%)	Bias	MSE	RE (%)	Bias	MSE	RE (%)
*n* = 200															
OLS	0.2530	0.0640	29.3	0.2505	0.0628	16.2	0.2489	0.0620	5.6	0.2503	0.0627	5.4	0.2492	0.0621	3.6
NTSLS	0.2801	0.0785	23.9	0.2816	0.0793	12.8	0.2864	0.0820	4.2	0.2854	0.0814	4.2	0.2865	0.0821	2.7
Median	0.2091	0.0439	42.7	0.1865	0.0348	29.2	0.1413	0.0200	17.5	0.1253	0.0157	21.7	0.1009	0.0102	22.0
Mode	0.0963	0.0244	77.0	0.0629	0.0132	76.9	0.0561	0.0058	59.7	0.0566	0.0051	66.4	0.0538	0.0035	64.8
LIVE	0.2555	0.0653	28.7	0.2217	0.0491	20.7	0.1623	0.0263	13.2	0.1396	0.0195	17.5	0.1145	0.0131	17.1
BSIVE	0.1199	0.0188	100	0.0887	0.0102	100	0.0476	0.0035	100	0.0497	0.0034	100	0.0415	0.0022	100
OTSLS	0.0106	0.0050		0.0150	0.0031		0.0135	0.0017		0.0194	0.0015		0.0159	0.0009	
*n* = 500															
OLS	0.2472	0.0611	7.0	0.2499	0.0624	3.5	0.2487	0.0618	2.2	0.2503	0.0626	1.7	0.2499	0.0625	1.2
NTSLS	0.2881	0.0830	5.1	0.2906	0.0844	2.6	0.2873	0.0825	1.7	0.2870	0.0824	1.3	0.2882	0.0830	0.9
Median	0.1441	0.0208	20.6	0.1197	0.0144	15.0	0.0870	0.0076	18.2	0.0797	0.0064	16.5	0.0624	0.0039	19.7
Mode	0.0315	0.0081	52.4	0.0304	0.0052	41.6	0.0283	0.0027	51.3	0.0309	0.0023	46.6	0.0305	0.0014	55.0
LIVE	0.1759	0.0309	13.8	0.1391	0.0194	11.2	0.0996	0.0099	13.9	0.0861	0.0074	14.2	0.0681	0.0046	16.5
BSIVE	0.0333	0.0043	100	0.0226	0.0022	100	0.0233	0.0014	100	0.0218	0.0011	100	0.0195	0.0008	100
OTSLS	0.0060	0.0024		0.0058	0.0012		0.0075	0.0008		0.0065	0.0006		0.0059	0.0003	
*n* = 1000															
OLS	0.2484	0.0617	2.6	0.2513	0.0632	1.8	0.2496	0.0623	1.2	0.2507	0.0628	0.8	0.2502	0.0626	0.7
NTSLS	0.2893	0.0837	1.9	0.2900	0.0841	1.3	0.2887	0.0834	0.9	0.2858	0.0817	0.6	0.2886	0.0833	0.5
Median	0.1014	0.0103	15.8	0.0842	0.0071	16.0	0.0630	0.0040	19.2	0.0507	0.0026	20.5	0.0440	0.0019	21.6
Mode	0.0177	0.0038	42.6	0.0189	0.0027	41.6	0.0138	0.0015	51.1	0.0182	0.0009	56.2	0.0213	0.0007	59.3
LIVE	0.1213	0.0147	11.1	0.0982	0.0097	11.8	0.0705	0.0050	15.4	0.0597	0.0036	14.8	0.0472	0.0022	18.8
BSIVE	0.0117	0.0016	100	0.0137	0.0011	100	0.0147	0.0008	100	0.0139	0.0005	100	0.0144	0.0004	100
OTSLS	0.0039	0.0012		0.0050	0.0007		0.0039	0.0003		0.0024	0.0002		0.0052	0.0002	

Note: RE (%) represents the relative efficiency and is calculated by dividing the MSE of any estimator by that of the BSIVE. Oracle-TSLS (OTSLS) is the estimator used when we precisely know which instruments are valid and which ones are not. The performance of the BSIVE approaches that of OTSLS.

**Table 3:** Monte Carlo simulation results for estimation performance with ρ=0.50,L=10 and r=3.

Method	SNR = 0.10			SNR = 0.25			SNR = 1			SNR = 2			SNR = 6		
	Bias	MSE	RE (%)	Bias	MSE	RE (%)	Bias	MSE	RE (%)	Bias	MSE	RE (%)	Bias	MSE	RE (%)
*n* = 200															
OLS	0.2494	0.0622	31.0	0.2511	0.0631	19.5	0.2503	0.0626	7.5	0.2515	0.0632	6.4	0.2505	0.0627	4.9
NTSLS	0.2812	0.0791	24.3	0.2811	0.0790	15.5	0.2744	0.0753	6.3	0.2733	0.0747	5.4	0.2769	0.0767	4.0
Median	0.2547	0.0650	29.6	0.2288	0.0525	23.4	0.1770	0.0313	15.1	0.1481	0.0219	18.3	0.1216	0.0148	20.8
Mode	0.1445	0.0373	51.6	0.1110	0.0215	57.1	0.0695	0.0094	50.1	0.0638	0.0060	67.5	0.0635	0.0045	68.4
LIVE	0.2458	0.0604	31.9	0.2095	0.0439	28.0	0.1463	0.0214	22.0	0.1242	0.0154	26.1	0.0990	0.0098	31.3
BSIVE	0.1217	0.0193	100	0.0980	0.0123	100	0.0613	0.0047	100	0.0537	0.0040	100	0.0514	0.0031	100
OTSLS	0.0148	0.0046		0.0151	0.0028		0.0122	0.0013		0.0107	0.0011		0.0106	0.0006	
*n* = 500															
OLS	0.2475	0.0612	7.8	0.2517	0.0634	3.7	0.2509	0.0629	2.5	0.2507	0.0629	2.0	0.2506	0.0628	1.9
NTSLS	0.2769	0.0767	6.2	0.2754	0.0759	3.1	0.2780	0.0773	2.0	0.2780	0.0773	1.7	0.2781	0.0773	1.6
Median	0.1796	0.0323	14.7	0.1484	0.0220	10.5	0.1156	0.0134	11.5	0.0993	0.0099	13.0	0.0802	0.0064	18.7
Mode	0.0460	0.0134	35.5	0.0422	0.0077	30.2	0.0375	0.0040	38.6	0.0408	0.0031	41.1	0.0399	0.0022	54.9
LIVE	0.1619	0.0262	18.2	0.1228	0.0151	15.4	0.0915	0.0084	18.5	0.0779	0.0061	21.1	0.0623	0.0039	30.9
BSIVE	0.0385	0.0048	100	0.0296	0.0023	100	0.0278	0.0015	100	0.0277	0.0013	100	0.0282	0.0012	100
OTSLS	0.0010	0.0015		0.0019	0.0010		0.0048	0.0006		0.0042	0.0004		0.0054	0.0002	
*n* = 1000															
OLS	0.2495	0.0622	2.7	0.2522	0.0636	1.7	0.2498	0.0624	1.2	0.2509	0.0629	1.1	0.2501	0.0625	0.9
NTSLS	0.2796	0.0782	2.2	0.2768	0.0766	1.4	0.2776	0.0771	1.0	0.2789	0.0778	0.9	0.2786	0.0776	0.7
Median	0.1352	0.0183	9.2	0.1151	0.0132	8.4	0.0837	0.0070	10.8	0.0707	0.0050	13.7	0.0569	0.0032	17.5
Mode	0.0331	0.0071	23.7	0.0225	0.0042	26.3	0.0294	0.0024	31.4	0.0281	0.0017	39.8	0.0268	0.0011	50.5
LIVE	0.1122	0.0126	13.4	0.0879	0.0077	14.4	0.0632	0.0040	19.0	0.0548	0.0030	22.7	0.0420	0.0018	32.0
BSIVE	0.0194	0.0017	100	0.0155	0.0011	100	0.0195	0.0008	100	0.0191	0.0007	100	0.0169	0.0006	100
OTSLS	0.0034	0.0007		0.0007	0.0005		0.0030	0.0003		0.0036	0.0002		0.0022	0.0001	

Note: RE (%) represents the relative efficiency and is calculated by dividing the MSE of any estimator by that of the BSIVE. Oracle-TSLS (OTSLS) is the estimator used when we precisely know which instruments are valid and which ones are not. The performance of the BSIVE approaches that of OTSLS.

**Table 4:** Monte Carlo simulation results for estimation performance with ρ=0.75,L=10 and r=3.

Method	SNR = 0.10			SNR = 0.25			SNR = 1			SNR = 2			SNR = 6		
	Bias	MSE	RE (%)	Bias	MSE	RE (%)	Bias	MSE	RE (%)	Bias	MSE	RE (%)	Bias	MSE	RE (%)
*n* = 200															
OLS	0.2498	0.0624	24.4	0.2487	0.0618	16.9	0.2499	0.0625	13.0	0.2490	0.0620	9.0	0.2501	0.0626	8.7
NTSLS	0.2798	0.0783	19.4	0.2748	0.0755	13.8	0.2745	0.0753	10.8	0.2763	0.0763	7.3	0.2779	0.0772	7.1
Median	0.2818	0.0818	18.6	0.2648	0.0710	14.7	0.2256	0.0509	15.9	0.2054	0.0422	13.2	0.1772	0.0314	17.4
Mode	0.1975	0.0581	26.2	0.1474	0.0397	26.3	0.1139	0.0177	45.7	0.0979	0.0131	42.7	0.0931	0.0097	56.3
LIVE	0.2440	0.0596	25.6	0.2028	0.0411	25.4	0.1496	0.0224	36.2	0.1239	0.0154	36.4	0.1116	0.0125	43.8
BSIVE	0.1092	0.0152	100.0	0.0948	0.0105	100	0.0826	0.0081	100	0.0705	0.0056	100	0.0703	0.0055	100
OTSLS	0.0076	0.0033		0.0101	0.0019		0.0090	0.0011		0.0082	0.0007		0.0089	0.0004	
*n* = 500															
OLS	0.2510	0.0630	10.1	0.2510	0.0630	6.1	0.2492	0.0621	3.5	0.2507	0.0629	2.9	0.2490	0.0620	3.3
NTSLS	0.2765	0.0764	8.4	0.2781	0.0773	5.0	0.2783	0.0775	2.8	0.2761	0.0762	2.4	0.2766	0.0765	2.7
Median	0.2520	0.0635	10.1	0.2058	0.0424	9.1	0.1649	0.0272	7.9	0.1405	0.0197	9.1	0.1163	0.0135	15.2
Mode	0.1166	0.0313	20.4	0.0650	0.0148	26.0	0.0675	0.0079	27.4	0.0616	0.0058	30.8	0.0614	0.0041	49.8
LIVE	0.1661	0.0276	23.2	0.1316	0.0173	22.3	0.0963	0.0093	23.2	0.0814	0.0066	27.2	0.0685	0.0047	43.9
BSIVE	0.0633	0.0064	100	0.0463	0.0039	100	0.0385	0.0022	100	0.0361	0.0018	100	0.0394	0.0021	100
OTSLS	0.0066	0.0013		0.0036	0.0009		0.0039	0.0004		0.0032	0.0003		0.0046	0.0002	
*n* = 1000															
OLS	0.2506	0.0628	3.6	0.2496	0.0623	1.9	0.2502	0.0626	1.4	0.2502	0.0626	1.2	0.2501	0.0625	1.4
NTSLS	0.2776	0.0771	2.9	0.2783	0.0774	1.5	0.2775	0.0770	1.2	0.2774	0.0769	1.0	0.2778	0.0772	1.2
Median	0.1889	0.0357	6.4	0.1563	0.0244	4.9	0.1191	0.0142	6.4	0.1024	0.0105	7.4	0.0841	0.0071	12.7
Mode	0.0462	0.0136	16.7	0.0456	0.0082	14.6	0.0450	0.0044	20.7	0.0405	0.0031	25.2	0.0424	0.0023	38.2
LIVE	0.1147	0.0132	17.3	0.0915	0.0084	14.3	0.0688	0.0047	19.2	0.0560	0.0031	24.8	0.0467	0.0022	41.1
BSIVE	0.0247	0.0023	100	0.0188	0.0012	100	0.0242	0.0009	100	0.0213	0.0008	100	0.0247	0.0009	100
OTSLS	0.0014	0.0006		0.0018	0.0004		0.0021	0.0002		0.0022	0.0001		0.0024	0.0001	

Note: RE (%) represents the relative efficiency and is calculated by dividing the MSE of any estimator by that of the BSIVE. Oracle-TSLS (OTSLS) is the estimator used when we precisely know which instruments are valid and which ones are not. The performance of BSIVE approaches that of OTSLS.

**Table 5:** Monte Carlo simulation results for estimation performance with L=40 and r=12.

Method	*ρ* = 0			*ρ* = 0.25			*ρ* = 0.50			*ρ* = 0.75		
	Bias	MSE	RE (%)	Bias	MSE	RE (%)	Bias	MSE	RE (%)	Bias	MSE	RE (%)
*n* = 200												
OLS	0.2524	0.0637	5.9	0.2502	0.0626	8.4	0.2622	0.0687	8.3	0.2667	0.0711	6.1
NTSLS	0.2947	0.0869	4.3	0.2952	0.0871	6.0	0.2864	0.0820	7.0	0.2844	0.0809	5.4
Median	0.1742	0.0304	12.3	0.2026	0.0410	12.8	0.2391	0.0572	10.0	0.2857	0.0816	5.3
Mode	0.0611	0.0057	65.0	0.0766	0.0091	57.6	0.1136	0.0209	27.4	0.2008	0.0546	7.9
LIVE	0.2132	0.0455	8.2	0.1838	0.0338	15.5	0.1426	0.0203	28.1	0.1123	0.0126	34.3
BSIVE	0.0554	0.0037	100	0.0667	0.0052	100	0.0681	0.0057	100	0.0623	0.0043	100
OTSLS	0.0292	0.0016		0.0206	0.0012		0.0044	0.0006		0.0030	0.0007	
*n* = 500												
OLS	0.2477	0.0613	1.0	0.2519	0.0634	0.6	0.2585	0.0668	1.4	0.2509	0.0629	2.8
NTSLS	0.2994	0.0897	0.7	0.2954	0.0872	0.4	0.2921	0.0853	1.1	0.2823	0.079	2.2
Median	0.1132	0.0128	4.9	0.1142	0.0130	2.8	0.1703	0.0290	3.2	0.2368	0.0561	3.2
Mode	0.0325	0.0030	21.0	0.0417	0.0043	8.6	0.0622	0.0052	18.0	0.1129	0.0219	8.1
LIVE	0.1423	0.0202	3.1	0.1111	0.0123	3.0	0.0957	0.0092	10.2	0.0710	0.0050	35.2
BSIVE	0.0182	0.0006	100	0.0067	0.0004	100	0.0259	0.0009	100	0.0365	0.0018	100
OTSLS	0.0124	0.0006		0.0017	0.0002		0.0055	0.0003		0.0020	0.0002	
*n* = 1000												
OLS	0.2506	0.0628	0.7	0.2526	0.0638	0.4	0.2445	0.0598	0.5	0.2509	0.0629	0.7
NTSLS	0.2954	0.0873	0.5	0.2934	0.0861	0.3	0.2913	0.0849	0.3	0.2824	0.0797	0.5
Median	0.0790	0.0062	7.0	0.0869	0.0076	3.8	0.1162	0.0135	2.1	0.1987	0.0395	1.1
Mode	0.0233	0.0014	31.2	0.0317	0.0018	15.8	0.0295	0.0026	10.5	0.0453	0.0088	4.9
LIVE	0.0954	0.0091	4.8	0.0816	0.0067	4.3	0.0668	0.0045	6.2	0.0511	0.0026	16.8
BSIVE	0.0110	0.0004	100	0.0071	0.0003	100	0.0062	0.0003	100	0.0148	0.0004	100
OTSLS	0.0046	0.0003		0.0064	0.0002		0.0031	0.0003		0.0013	0.0001	

**Table 6:** Empirical findings: The effects of depression and BMI on HRQLI

Method	Depression†		BMI‡	
	$\hat{\beta }$	95% Confidence Interval	$\hat{\beta }$	95% Confidence Interval
OLS	−0.0066	(−0.0073, −0.0059)	−0.0074	(−0.0092, −0.0055)
NTSLS	−0.0063	(−0.0141, 0.0016)	−0.0071	(−0.0168, 0.0025)
Median	−0.0070	(−0.0266, 0.0126)	−0.0090	(−0.0247, 0.0067)
Mode	−0.0050	(−0.0148, 0.0048)	−0.0110	(−0.0483, 0.0263)
LIVE	−0.0060	(−0.0174, 0.0054)	−0.0069	(−0.0168, 0.0031)
BSIVE	−0.0047	(−0.0135, 0.0041)	−0.0052	(−0.0175, 0.0071)

Note: First-stage regression results:

† F-test = 2.32;

‡ F-test = 1.43.

## Footnotes

1 ”The objective of Mendelian randomization is to assess the causal impact of an exposure on an outcome variable using genetic variants as instruments.” For further guidance on analyzing Mendelian randomization, refer to Burgess et al. (2023).

2 Note that we use a two-step procedure similar to that in Kang et al. (2016). In the first step, we solve the best-subset regression problem by using MIO with modifications made to the variables $\dot{Y}$ and $\dot{Z}$, thereby estimating $\overset{ˆ}{\delta }$ via Algorithm 1. In the second step, we use the estimated $\overset{ˆ}{\delta }$ to solve (5.1) for β, which is as computationally efficient as the least-squares problem. The two-step procedure yields the same solution as that of the best-subset problem in (3.1). This estimation procedure is very useful, as datasets often have substantial size and complexity.

3 Normally, “two-sample methods can be used when a set of instruments is common to two data sets but endogenous and dependent variables are included in only one or the other data set” (Angrist & Krueger, 1992). Median and mode estimators are types of two-sample IV methods specifically used for Mendelian randomization analysis. These methods are particularly useful when only the regression coefficient and its standard error for gene-exposure and gene-outcome associations are available from separate studies. However, two-sample methods may also be applied to one-sample data (Minelli et al., 2021).

4 The Lasso estimates are nonlinear and nondifferentiable functions of the outcome values, and therefore, obtaining accurate estimations of their standard errors is difficult. As an alternative, Tibshirani (1996) suggested the use of bootstrapping to calculate the standard error. Bootstrapping methods are commonly used in statistics and econometrics, as well as in Mendelian randomization (see, e.g., Aikens et al., 2017 and Burgess et al., 2015). Therefore, the standard error and confidence intervals of the BSIVE and LIVE are calculated by bootstrapping with 500 resamples.

5 ”Disease/behavior” of SNPs: rs1800497 “nicotine/alcohol addiction”; rs2242592 “impulsivity and Parkinson’s”; rs1421085 “obesity, Alzheimer’s, diabetes”; rs6314 “memory” and rs6265 “cognitive aging, memory, intelligence quotient”. More information about SNPs is available on the website https://www.ncbi.nlm.nih.gov/snp/.
